# Barriers and misconceptions hindering reduction of intestinal schistosomiasis in Mbita Sub-County, Western Kenya

**DOI:** 10.1186/s41182-024-00602-7

**Published:** 2024-05-14

**Authors:** Ngetich B. Cheruiyot, Sachiyo Nagi, Asena E. Chadeka, Rie Takeuchi, Miho Sassa, Bahati Felix, Noriko Kobayashi, Taeko Moriyasu, Janet Masaku, Gordon Okomo, Collins Ouma, Doris Njomo, Sammy M. Njenga, Shinjiro Hamano

**Affiliations:** 1 Nagasaki University, Institute of Tropical Medicine (NUITM): Kenya Medical Research Institute (KEMRI) Project, P O Box 19993-00202, Nairobi, Kenya; 2 Department of Hygiene and Public Health, Tokyo Women’s Medical University, 8-1 Kawada-Machi, Shinjuku-ku, Tokyo, 162-0054 Japan; 3 Department of Parasitology, Institute of Tropical Medicine (NEKKEN), Nagasaki University, 1-12-4 Sakamoto, Nagasaki, 852-8523 Japan; 4 Graduate School of Public Health, International University of Health and Welfare, 4-3, Kodunomori, Narita, Chiba, 286-8686 Japan; 5 Department of Global Health Policy, Graduate School of Medicine, The University of Tokyo, Tokyo, Japan; 6 Eastern and Southern Africa Centre of International Parasite Control (ESACIPAC), Kenya Medical Research Institute (KEMRI), Nairobi, Kenya; 7 Department of Health Services, County Government of Homa Bay, Homa Bay, Kenya; 8 Department of Biomedical Sciences and Technology, School of Public Health and Community Development, Maseno University, Kisumu, Kenya

## Abstract

**Background:**

Community and individual participation are crucial for the success of schistosomiasis control. The World Health Organization (WHO) has highlighted the importance of enhanced sanitation, health education, and Mass Drug Administration (MDA) in the fight against schistosomiasis. These approaches rely on the knowledge and practices of the community to be successful; however, where the community knowledge is low and inappropriate, it hinders intervention efforts. Hence, it is essential to identify barriers and misconceptions related to awareness of schistosomiasis, sources of infection, mode of transmission, symptoms, and control measures.

**Methods:**

This was a mixed-method cross-sectional study involving 1200 pre-school children randomly selected and examined for *Schistosoma mansoni* infection using the Kato-Katz technique. All parents/guardians of selected children were enrolled for a pre-tested questionnaire survey, while 42 were engaged in focus group discussions (FGDs). The level of knowledge and awareness among parents/guardians about schistosomiasis was evaluated in relation to the infection status of their pre-school children.

**Results:**

Among pre-school children, the prevalence of intestinal schistosomiasis was 45.1% (95% CI 41.7–48.5). A majority of parents/guardians (85.5%) had heard about schistosomiasis, and this awareness was associated with the participant’s level of education (OR = 0.16, 95% CI 0.08, 0.34). In addition, a positive association was observed between higher educational attainment and knowledge of the causative agent (OR = 0.69, 95% CI 0.49, 0.96). Low education level was significantly associated with limited knowledge of transmission through lake water contact (OR = 0.71, 95% CI 0.52, 0.97) and infection from the lake (OR = 0.33, 95% CI 0.19, 0.57). Notably, parents/guardians who have heard of schistosomiasis could not recognize symptoms of *S. mansoni* infection, such as abdominal pain (91.8%, 815/888) and blood in the stool (85.1%, 756/888). Surprisingly, 49.8% (442/888) incorrectly identified hematuria (blood in urine), a key sign of *S. haematobium,* but not *S. mansoni,* in an endemic area for *S. mansoni* infection. The majority (82.6%, 734/888) of parents/guardians were unaware that dams are potential infection sites, despite 53.9% (479/888) of their pre-school-aged children testing positive for schistosome infection.

**Conclusions:**

Despite the high level of awareness of intestinal schistosomiasis in the study area, we identified a low level of knowledge regarding its causes, modes of transmission, signs and symptoms and potential sites of transmission within the community. This study emphasizes the need for targeted educational interventions to address the misconceptions and knowledge gaps surrounding intestinal schistosomiasis through tailored community-based programs.

## Introduction

Schistosomiasis is the second most prevalent parasitic disease after malaria in low- and middle-income countries, particularly in Sub-Saharan Africa [1, 2]. It is estimated that over 779 million people are at risk of schistosome infection [3, 4], and pre-school children have been reported to suffer the affliction of schistosomiasis [5–7]. The transmission cycle of schistosome involves the contamination of fresh surface water with infected human waste. The parasite eggs within the human waste hatch and release miracidia, which are able to penetrate and infect freshwater snails of the genus *Biomphalaria for Schistosoma mansoni* and the genus *Bulinus for Schistosoma haematobium* [8]. Infected snails, acting as intermediate hosts, release cercariae into freshwater sources, which are able to penetrate the human skin when individuals come into contact with contaminated water sources [9]. It is, therefore, endemic in resource-poor settings characterized by lack of potable water and poor sanitation.

In Kenya, intestinal schistosomiasis caused by *S. mansoni* infection is widely distributed in areas along the shores of Lake Victoria. On the other hand, urogenital schistosomiasis caused by *S. haematobium* is prevalent along the Kenyan coast [10]. Although the overall prevalence of schistosome infection is relatively high in the Western part of Kenya, it varies based on age group. A prevalence of 3.6% was reported for children under 2 years, 45.1% for pre-school children, and varying rates for primary school pupils, ranging from 60% to 76.8% depending on the locations of the primary schools and the time of study [11–14]. Specific knowledge on the causes, mode of transmission and signs and symptoms that contribute to its prevention and control is inadequate among communities [15, 16]. The level of knowledge varies from 50% to 90% depending on the community setting [16–18]. The inadequate knowledge has perpetuated confusion about schistosome infection causes with those of soil-transmitted helminths (STH) and even in its perceived control measures [19–21]. The signs and symptoms of intestinal schistosomiasis are mainly abdominal pain and bloody stool, but urogenital schistosomiasis is characterized by haematuria (blood in urine) as the classical sign [22]. Confusion and misunderstanding of critical signs and symptoms specific to *S. mansoni* infection are barriers towards realizing the potential benefits of transmission interruption for schistosome infection [23]. Recognition of symptoms and causes of schistosomiasis that are inconsistent with the disease have also been reported in other studies [16, 24].

Schistosomiasis control efforts advocated by the World Health Organization (WHO) revolve around four fundamental approaches: preventive chemotherapy, water sanitation and hygiene interventions, environmental interventions, health education and behaviour change communication [25]. The WHO further recommends the integration of schistosomiasis education into the routine activities of health facilities. There is a need in improving global strategy from morbidity control through reduction to elimination of schistosomiasis [26]. Mass drug administration (MDA) has significantly reduced morbidity, but reinfection occurrence reverses the achievements. Schistosomiasis education as a capacity- and awareness-building component provides an excellent avenue to complement MDA in its control. Correct and comprehensive knowledge has been proven to influence protective behaviour and health attitudes positively [17, 27], and it is disastrous if the community is limited in understanding the causes, sources, transmission and prevention of schistosomiasis.

Clarifying the knowledge and practices of at-risk populations is crucial for suitable schistosomiasis interventions. Equally important is identifying misconceptions that are barriers to the control and prevention of schistosomiasis. The present study was, therefore, undertaken to understand existing knowledge and misunderstandings of pre-school parents/guardians regarding intestinal schistosomiasis, its source of infection, mode of transmission, causes, symptoms, treatment and control.

## Methods

### Study area

**Fig. 1:**
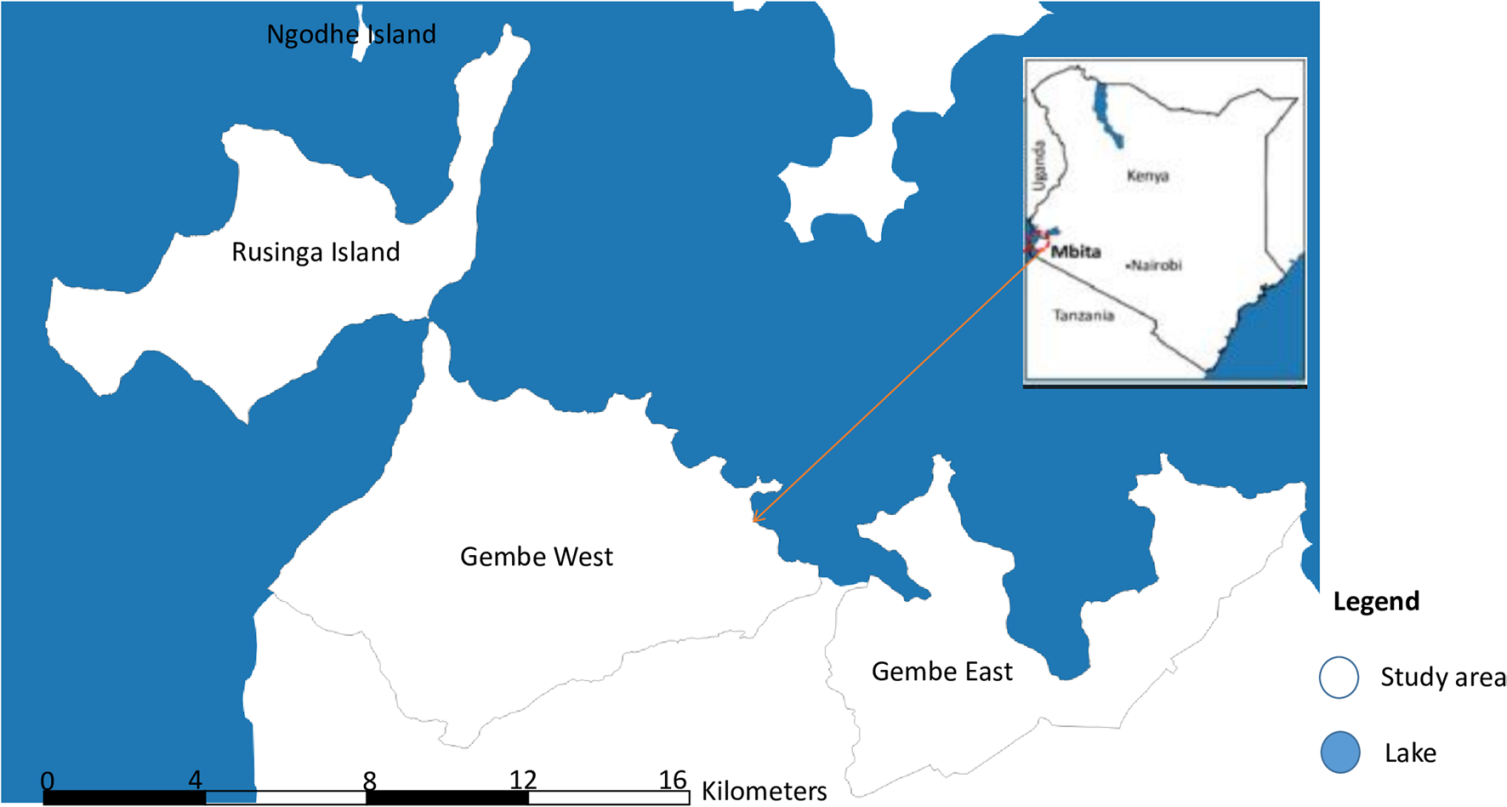
Map of the study area. The map was developed using QGIS version 11.7 software, and the shape files were sourced from https://www.igismap.com/kenya-shapefile-download-boundary-line-administrative-state-and-polygon/

The study was conducted in Mbita Sub-County of Homa Bay County in western Kenya (Fig. 1). The area is under the Health Demographic Surveillance System (HDSS). It covers four locations: Rusinga East and West on the island of Lake Victoria and Gembe East and West along the lake shores of the mainland. According to the Kenya Population and Housing Census (KPHC) 2019, Mbita HDSS area had a total population of 68,319 [28]. Most residents belong to the Luo ethnic group and language. Their main economic activities are fishing and small businesses, especially along the lake.

Majority of the residents depend on Lake Victoria as their primary source of water and the area reported a toilet coverage of 42% [29]. The temperature ranges from 15 °C to 30 °C, while rain seasonality is bimodal, with the long rain season from March to May and short rain season from October to December. Estimated annual rainfall ranges between 800 and 1900 mm in the study area. The area is served by one sub-county hospital, two health centres and five dispensaries. In addition, private clinics and pharmacies are common in Mbita town. Access to basic education in the county was slightly better than the national status in 2015, using Net Enrolment Ratio (NER) indicator. The NER progress for pre-primary, primary and secondary was 99.4%, 97.3% and 55.1%, respectively [30].

### Study design

A mixed-method cross-sectional study was conducted among pre-school children and their parents/guardians. A total of 66 pre-schools were randomly selected from 142 pre-school centers for participation in the study. From a population of 5580 pre-school children, 1200 participants were sampled proportionately from the 66 selected pre-schools using a sample size formula for finite population [31]. The selected pre-school children participated in a parasitological study from September to October 2014 [13]. A questionnaire survey was conducted between January and February 2015 among 1200 parents/guardians purposively selected from all the chosen pre-school children. The questionnaires were originally written in English and then translated into Dholuo. A sample of these parents/guardians was later enrolled for focus group discussions (FGDs). The questionnaire was designed to capture information on the parents/guardian’s demographic characteristics and knowledge about schistosomiasis. The questionnaire also captured the source of knowledge, perceived cause, source of infection, mode of transmission, signs and symptoms. Participants in the qualitative study were organized into four focus groups based on the four administrative units within the Mbita HDSS area. Gembe East (group 1) on the mainland had 11 participants, Gembe West (group 2) also on the mainland comprised 9 members, and on the island of Rusinga, 13 participants and 9 participants attended the FGDs in Rusinga West (group 3) and East (group 4), respectively. The participants were randomly selected from a list of parents/guardians who participated in the household questionnaire survey. All FGDs were facilitated in the local language (*Dholuo*) by trained personnel from the local community and conducted according to the interview guide, which included questions on the mode of transmission, source of infection, and information source. All the notes and audio records were coded and later transcribed and translated into English by two independent officers. Finally, a back translation was performed to ensure a unified meaning of transcripts and minimize bias. The study assessed the knowledge and awareness of parents/guardians regarding schistosomiasis in relation to the infection status of pre-school children.

### Parasitological stool assessment

Stool samples were collected for two consecutive days from sampled pre-school children after prior delivery of labelled stool containers. During stool collection, the sample was adequately coded and transported for examination. *S. mansoni* quantification was performed using the Kato-Katz faecal thick smear technique [32]. Each stool sample was processed in duplicate, and the slide pair was examined within an hour for the presence of eggs by two independent laboratory technologists. Any discrepancy in results was reconciled by a senior microscopist. *S. mansoni* infection intensity was based on egg per gram of stool (epg) and was classified as either light (< 100 epg), moderate (100–399 epg), or heavy (≥ 400 epg) as previously described [33].

### Data management and analysis

Quantitative data collected were double-entered, cross-checked using Microsoft Excel (2007 version) and exported for analysis in R [34]. The association test was done first at a bivariate level, and a multivariate model was later fitted for variables below a significance level of 0.05 in order to adjust for covariates. The Chi-square test and Fisher’s exact test were applied appropriately to assess the association among parents/guardians’ knowledge, awareness, education level and infection status of their children. Qualitative data from the FGDs were transcribed and translated then written responses were analyzed thematically according to emerging study themes and sub-themes. The themes from the text [35] were developed into cohesive ideas and presented in text format. Participant's direct quotes were added to illustrate the text.

The themes included the assessment of participant’s knowledge and awareness of schistosomiasis causes, transmission, signs and symptoms, and control.

## Results

### Prevalence and intensity

Parasitological information for pre-school children showed an overall prevalence of 45.1% (95% CI 41.7–48.5) for *S. mansoni* infection. The intensity and associated high transmission areas have already been published [13].

### Demographic characteristics of the respondents

A total of 1038 pre-school children from 66 schools with complete data were considered for analysis. The age range of the examined pre-school children was 2–9 years, with a mean age of 5.7 ± 1.5 years. Out of these children, 512 (49.3%) were females and 526 (50.7%) were males. The average age of the parents/guardians of the enrolled children was 33.4 ± 10.8 years, with the majority being women (83.3%). Most of the respondents were married (83.0%) and had, at most, a primary level of education (77.2%). Lake water (91.3%) remained the primary source for all domestic purposes within this community (Table 1).

**Table 1:** Sociodemographic characteristics of the pre-school children and parents/guardians

Variables		*n* (%)
Pre-school children (*n* = 1038)		
Age (years)	Range	2 to 9
	Mean	5.7
	Standard deviation	± 1.5
Sex	Girls	512 (49.3)
	Boys	526 (50.7)
Parents/guardians (*n* = 1038)		
Age (years)	Range	19 to 83
	Mean	33.51
	Standard deviation	±10.72
Age group	< 25	161 (15.5)
	25–34	522 (50.3)
	35–44	211 (20.3)
	45–54	80 (7.7)
	> 54	64 (6.2)
Age (years)	Mean	33.4
	Standard deviation	± 1.8
Sex	Female	865 (83.3)
	Male	173 (16.7)
Marital status	Married	862 (83.0)
	Single	37 (3.6)
	Widowed	139 (13.4)
Education level	Primary and below	801 (77.2)
	Secondary and above	237 (22.8)
Main source of water	Lake	948 (91.3)
	Dam	27 (2.6)
	Spring	44 (4.2)
	Tap	19 (1.9)
Parents/guardians FGD Participants (*n* = 42)		
Age (years)	Range	19 to 75
	Mean	35.64
	Standard deviation	± 12.7
Sex	Female	34 (81.0)
	Male	8(19.0)
Education Level	Primary	19 (45.2)
	Secondary	18 (42.9)
	Tertiary	5 (11.9)

FGD: focus group discussion

### Awareness about *S. mansoni* infection among parents/guardians of pre-school children

The majority of the respondents (85.5%) had heard about schistosomiasis through various sources of information; media (38.9%) and health facilities (31.6%) were the major sources. Other sources of information included friends/relatives (28.5%), school (27.9%), research programs (25.7%) and community *baraza* (3.0%), which is a public gathering meant for raising awareness and sharing knowledge to the community members. Refer to Table 2.

**Table 2:** Sources of schistosomiasis information among parents/guardians

Variables	*n* (%)
Parents/guardians (n = 888)	
Media (Radio, TV, newspapers)	345 (38.9)
Health facility	281 (31.6)
Friends/Relatives	253 (28.5)
School	248 (27.9)
Research Programs	228 (25.7)
Community *baraza*	27 (3.0)

During FGDs, several misconceptions regarding the transmission of schistosomiasis were revealed. For instance, some participants believed that individuals who urinated in the lake infested with schistosomes could infect themselves during urination. Similarly, other reported misconceptions included drinking untreated water, consuming contaminated food, or even getting pricked by a thorn in the lake.

“*…..when we urinate in the lake that contains bilharzia, we get bilharzia*” (30-year-old business woman).

“*…..It can be found when one urinates in the lake or when house fly carries faeces and lands on your food with those worms*.” (39-year-old businessman).

“*…..people contact when they drink water containing bilharzia worms, and the worms enter the body*” (23-year-old housewife).

However, some participants noted positive responses, such as engaging in activities like playing and bathing in infested lake water.

“*…..bilharzia is contracted when we bathe in the lake or the river.*” (32-year-old madam teacher).

Contributions from FGDs brought significant views on blood in urine as a sign of schistosomiasis as a disease. Furthermore, upon probing on signs specific to intestinal schistosomiasis, participants demonstrated low awareness, although they could mention abdominal pain, diarrhoea, malaise, swollen belly, itchy rashes and bloody stool. The discussion revealed a consensus among respondents that there were challenges in identifying signs and symptoms related to *S. mansoni* infection.

“ *….. we cannot identify whether we are suffering from bilharzias because we don’t know the symptoms in our body*” (27-year-old peasant farmer)

“…..*urinating is difficult for those who are infected with bilharzias because they feel pain while passing urine*” (38-year-old business lady)

“…..*the body of an infected person contains rashes that itch and their faeces contain blood*” (40-year-old fisherman)

Interactions during the FGDs highlighted that the local community commonly referred to schistosomiasis as *“layo remo”,* which translates to “urinating blood” as local language and is associated with *S. haematobium* infection.

Regarding the prevention and control of schistosomiasis, FGD participants demonstrated a lack of awareness about preventive measures. Many of those who understood the source of infection as the lake water believed, it is challenging to avoid contracting schistosomiasis since the lake is their primary water source. Others perceived wearing shoes will help in the prevention of infections.

“.….*people do not have enough information on bilharzias, so they have no idea how it is transmitted and how it can be controlled*” (39-year-old farmer).

“.….*most people cannot stop using the lake water, thus making it difficult to control bilharzia*.” (61-year-old housewife).

“.….*we can control it if children put on their shoes while walking on the lake shore or bushes*” (28-year-old housewife).

### Factors associated with schistosomiasis knowledge among parents/guardians of pre-school children

The education level of the respondent was a vital variable that was significantly associated with the knowledge and awareness variables. Respondents with primary education levels and below were less likely to have heard of schistosomiasis (OR = 0.16, 95% CI 0.08, 0.34). In addition, they were unlikely to understand the parasites (worm) as the causative agent compared to those with secondary education and beyond (OR = 0.69, 95% CI 0.49, 0.96).

Knowledge that contact with lake water is associated with transmission of schistosome infection was significantly lower among respondents with primary education (OR = 0.71, 95% CI 0.52, 0.97). Similarly, respondents with primary education level were less likely to identify the lake as a source of schistosome infection than those with secondary and higher level of education (OR = 0.33, 95% CI 0.19, 0.57) (Table 3).

**Table 3:** Association of knowledge of the respondents on schistosomiasis with education level

Variables		Education level			
		At most Primary	Secondary and above	COR	AOR
		*n* (%)	*n* (%)	95% CI	
Awareness (*n* = 1038)					
Heard of schistosomiasis	No	142 (17.7)	8 (3.4)	1.00	
	Yes	659 (82.3)	229 (96.6)	0.16 (0.08,0.34)	0.15 (0.07,0.30)
Cause (*n* = 888)					
Bacteria	No	611 (92.7)	205 (89.5)	1.00	
	Yes	48 (7.3)	24 (10.5)	0.67 (0.40, 1.12)	_
Virus	No	645 (97.9)	227 (99.1)	1.00	
	Yes	14 (2.1)	2 (0.9)	2.46 (0.66, 15.26)	_
Parasites (Worm)	No	224 (34.0)	60 (26.2)	1.00	
	Yes	435 (66.0)	169 (73.8)	0.69 (0.49, 0.96)	0.70 (0.49,0.97)
Mode of transmission (*n* = 888)					
Drinking water	No	339 (51.4)	101 (44.1)	1.00	
	Yes	320 (48.6)	128 (55.9)	0.74 (0.55, 1.01)	
Food	No	640 (97.1)	226 (98.7)	1.00	
	Yes	19 (2.9)	3 (1.3)	2.24 (0.65, 11.90)	
Lake water contact	No	302 (45.8)	86 (37.6)	1.00	
	Yes	357 (54.2)	143 (62.4)	0.71 (0.52, 0.97)	0.89 (0.53,0.98)
Sign and symptoms (*n* = 888)					
None	No	651 (98.8)	227 (99.1)	1.00	
	Yes	8 (1.2)	2 (0.9)	1.39 (0.29, 6.62)	_
Abdominal pain	No	602 (91.4)	213 (93.0)	1.00	
	Yes	57 (8.6)	16 (7.0)	0.26 (0.71, 2.24)	_
Blood in stool	No	570 (86.5)	186 (81.2)	1.00	
	Yes	89 (13.5)	43 (18.8)	0.68 (0.45,1.01)	_
Blood in urine	No	332 (50.4)	114 (49.8)	1.00	
	Yes	327 (49.6)	115 (50.2)	0.98 (0.72,1.32)	_
Stunting	No	642 (97.4)	225 (98.3)	1.00	
	Yes	17 (2.6)	4 (1.7)	1.49 (0.55, 4.86)	_
Source of infection (n = 888)					
Lake	No	116 (17.6)	15 (6.6)	1.00	
	Yes	543 (82.4)	214 (93.4)	0.33 (0.19, 0.57)	0.31 (0.17,0.53)
Dam	No	542 (82.2)	192 (83.8)	1.00	
	Yes	117 (17.8)	37 (16.2)	1.12 (0.75, 1.68)	_
Toilet	No	629 (95.5)	221 (96.5)	1.00	
	Yes	30 (4.5)	8 (3.5)	1.32 (0.60, 2.92)	_
Playground	No	649 (98.5)	224 (97.8)	1.00	
	Yes	10 (1.5)	5 (2.2)	0.69 (0.23, 2.04)	_

OR: Odds ratio, AOR: Adjusted Odds Ratio. CI: Confidence interval. *Significant association (*p* < 0.05). For the AOR, adjustments were made based on age and sex [14, 36]

A multivariate logistic regression analysis of factors significantly associated with knowledge while adjusting for age and sex revealed that participants with primary education level and below were less likely to have heard about schistosomiasis (OR = 0.15, 95% CI 0.07, 0.30). The same group of participants had significantly lower knowledge of parasite worms as the causative agent (OR = 0.70, 95% CI 0.49, 0.97). In addition, they had inadequate knowledge of lake water contact as a transmission mode (OR = 0.89, 95% CI 0.53, 0.98) and would least associate the lake with schistosome infection (OR 0.31, 95% CI 0.17, 0.53).

Table 4 shows parents/guardians, whose pre-school children tested positive for *S. mansoni* infection had more knowledge of lake water contact than those whose children tested negative (OR = 1.32, 95% CI 1.01, 1.72). However, parents/guardians of schistosome-infected children showed low knowledge of the dam as a potential source of schistosome infection (OR = 0.60, 95% CI 0.42, 0.86). Further multivariate logistic analysis while adjusting for age, sex and education of the respondents showed that parents/guardians, whose pre-school children were *S. mansoni* positive had higher knowledge about lake water contact as a mode of transmission (AOR = 1.35, 95% CI 1.03, 1.77). Conversely, they were less likely to identify the dam as a potential source of schistosome infection (AOR = 0.59, 95% CI 0.41, 0.85).

**Table 4:** Association of parents/guardians’ knowledge of schistosome infection status of their pre-school children

Variables	Infection status				
		Negative	Positive	COR	AOR
		*n* (%)	*n* (%)	95% CI	
Awareness (*n* = 1038)					
Heard of schistosomiasis	No	85 (15.2)	65 (13.6)		
	Yes	474 (84.8)	414 (86.4)	0.97 (0.60,1.57)	
Cause (*n* = 888)					
Bacteria	No	435 (91.8)	381(92.0)		
	Yes	39 (8.2)	33(8.0)	0.97 (0.60, 1.57)	
Virus	No	464 (97.9)	408(98.6)		
	Yes	10 (2.1)	6 (1.4)	0.68 (0.25, 1.89)	
Parasites (Worm)	No	158 (33.3)	126 (30.4)		
	Yes	316 (66.7)	288 (69.6)	1.14 (0.86, 1.52)	
Mode of transmission (*n* = 888)					
Drinking water	No	221 (46.6)	219 (52.9)		
	Yes	253 (53.4)	195 (47.1)	0.78 (0.60, 1.01)	
Food	No	466 (98.3)	400 (96.6)		
	Yes	8 (1.7)	14 (3.4)	2.04 (0.85, 4.91)	
Lake water contact	No	222 (46.8)	166 (40.1)		
	Yes	252 (53.2)	248 (59.9)	1.32 (1.01,1.72)*	1.35 (1.03,1.77)*
Sign and symptoms (*n* = 888)					
None	No	467 (98.5)	411 (99.3)		
	Yes	7 (1.5)	3 (0.7)	0.49 (0.11, 1.77)	
Abdominal pain	No	441 (93.0)	374 (90.3)		
	Yes	33 (7.0)	40 (9.7)	1.43 (0.88, 2.31)	
Blood in stool	No	404 (85.2)	352 (85.0)		
	Yes	70 (14.8)	62 (15.0)	1.02 (0.70, 1.47)	
Blood in urine	No	240 (50.6)	206 (49.8)		
	Yes	234 (49.4)	208 (50.2)	1.04 (0.80, 1.35)	
Stunting	No	461 (97.3)	406 (98.1)		
	Yes	13 (2.7)	8 (1.9)	0.70 (0.29, 1.70)	
Source of infection (*n* = 888)					
Lake	No	76 (16.0)	55 (13.3)		
	Yes	398 (84.0)	359 (86.7)	1.25 (0.86, 1.81)	
Dam	No	376 (79.3)	358 (86.5)		
	Yes	98 (21.7)	56 (13.5)	0.60 (0.42, 0.86)*	0.59 (0.41,0.85)*
Toilet	No	448 (94.5)	402 (97.1)		
	Yes	26 (5.5)	12 (2.9)	0.51 (0.26, 1.03)	_
Playground	No	464 (97.9)	409 (98.8)		
	Yes	10 (2.1)	5 (1.2)	0.57 (0.19, 1.56)	_

OR: Odds ratio, AOR: Adjusted Odds Ratio. CI: Confidence interval. *Significant association (*p* < 0.05). For the AOR, adjustments were made on age and sex based on a previous study [37]

## Discussion

The study findings recorded intestinal schistosomiasis prevalence of 45.1% (95% CI 41.7–48.5) among pre-school children, and this, according to WHO, is of public health importance and requires attention [25]. The objective of the present study was to assess barriers and misconceptions on intestinal schistosomiasis knowledge regarding its causes, modes of transmission, signs and symptoms and potential sites of transmission within the community among parents/guardians. The study observed majority of the respondents (85.5%) to have heard of schistosomiasis through varied sources but with inadequate knowledge on specific areas. These findings agreed with a previous study in Senegal [38], which reported that most of the study participants were aware of schistosomiasis but had inadequate knowledge about symptoms and modes of transmission.

Despite the high proportion of respondents reporting having heard of schistosomiasis, limited knowledge and misconceptions were exhibited on the understanding of the causes, mode of transmission and symptoms. A similar observation was noted in Zimbabwe [21]. There is a need to enhance community knowledge on causes to help demystify such misconceptions, considering the role friends and relatives play in knowledge transmission. Dawaki et al*.* [17] noted concerns about the dilution of knowledge information by family members and neighbours. However, another researcher [39] appreciated how friends and family pass helpful information towards reducing schistosomiasis infection. Ensuring communities get the correct information is vital to make them part and parcel of the control initiatives. The awareness of the causative agent was considerably low among respondents with primary education. This could be due to the limited nature and methods of transmitting health information messages. It, therefore, calls for targeted messages in local dialects and national languages to correct misconceptions arising in low levels of knowledge, as noted by earlier studies [40, 41]. Previous studies established poor knowledge/or misunderstanding about the transmission mode among communities. For example, a study conducted in Senegal reported an inadequate knowledge of the transmission mode despite health education programs [38], while other studies in western Kenya reported similar findings [15, 42].

Similarly, our study revealed low awareness of the transmission mode and further noted possible misconceptions, which concur with other studie*s.,* which have reported a low level of knowledge on transmission of schistosomiasis [15, 43–45]. Confusion was exhibited through responses like wearing shoes to prevent schistosome infection. In addition, participants believed they were infected with schistosome while urinating in the lake water. In as much as urinating in the lake water is associated with the cause of urogenital schistosomiasis, it is interesting that respondents in the *S. mansoni* endemic area could mention it. The perception that schistosome transmission occurs when urinating into a water body requires attention since such deviation from schistosome transmission is a potential hindrance to prevention and control [27]. Such a perception provides a false hope of safety from contracting schistosomiasis by not urinating in the lake water and contributes to increased transmission in the community [46].

Misconceptions on transmission mode were also evident among respondents with secondary education levels and beyond, as illustrated by their knowledge of associating transmission with drinking untreated water. This finding is consistent with a previous study around Lake Victoria, where participants also reported drinking untreated water as a mode of schistosome transmission [20]. This misconception may stem from their awareness of other waterborne diseases caused by consuming untreated water. The current study results further show that education level positively correlated with participants’ knowledge, as those with a secondary education level and above demonstrated a better understanding of schistosome transmission. People with a higher level of education can link contact with lake water to schistosomiasis, which is crucial for reducing the risk of infection and reinfection [17].

The study revealed that the education level of the respondents played a significant role in their understanding of the causes of schistosomiasis. Participants with a primary education level had limited knowledge and were less likely to mention worms as the causative agent of schistosome infection. This finding aligns with a previous study conducted in Uganda, which also identified education level as a significant factor influencing knowledge about schistosomiasis [19]. Despite the awareness demonstrated by some respondents, the lack of alternative water sources free from schistosome infections compelled them to rely on lake water. This highlights the challenges communities face in accessing safe and uncontaminated water sources, further contributing to the risk of schistosome transmission.

The community referred to schistosomiasis in the local dialect as *layo remo,* meaning urinating blood, with the name inclined to *S. haematobium* typical symptom [22]. Despite the high prevalence and intensity of intestinal schistosomiasis in Mbita Sub-County [12], respondents’ knowledge of signs and symptoms was low and misguided. A majority indicated heightened awareness of blood in urine compared to their understanding of blood in stool symptoms, though insignificant. The misunderstanding could stem from the local name *layo remo* in *Dholuo,* which translates to urinating blood. Nonetheless, the findings concurred with previous studies in Kenya [15, 42], in Cote d’Ivoire [38] and in Nigeria [17]. Other studies attributed low awareness of intestinal schistosomiasis to confusion with other diseases exhibiting similar symptoms [47, 48]. We attributed it to minimal awareness creation of intestinal schistosomiasis in all groups within the community. The fact that participants with higher education levels were more knowledgeable regarding the lake as a potential infection site demonstrates that they could benefit from the general health information.

Parents/guardians of pre-school children who tested positive for intestinal schistosomiasis showed awareness of lake water contact as a transmission mode. This awareness perhaps originated from the previous schistosomiasis history of their children and associated treatment. This finding suggests that informed parents/guardians may be more vigilant in monitoring and regulating their children's exposure to potentially contaminated water sources, thereby reducing the risk of *S. mansoni* infection. A study by Gryseels et al. [49] agrees with the finding that community-based health education programs targeting parents and caregivers were effective in increasing knowledge about schistosome transmission and prevention measures. The observed association between the parent awareness and the infection status of the pre-school child highlights the importance of targeted educational interventions aimed at enhancing parental awareness of schistosome transmission routes, particularly related to lake water contact. By empowering parents with accurate information and preventive strategies, public health efforts can potentially mitigate the burden of schistosomiasis in at-risk communities and protect the health of vulnerable populations, such as pre-school children.

Generally, the study participants demonstrated low awareness of the dam as a potential site of infection for schistosomiasis. They thus could exacerbate schistosomiasis infection with the assumption that they are safe water sources. Their overall awareness of prevention and control measures was low, as they acknowledged the difficulty in controlling or preventing schistosomiasis. Bold statements like “it is difficult to control schistosomiasis as long as we continue to use the lake water” from the participants highlight the challenge of changing their perception.

## Limitations

The research tools were only administered in English and Dholuo, potentially introducing bias. The Kato-Katz test was used without a complementary test, although stool samples were collected on consecutive days and analyzed in duplicate to minimize any bias.

## Conclusion

This study underscores the critical need for targeted educational interventions to address the pervasive misconceptions and gaps in knowledge surrounding intestinal schistosomiasis within affected communities. The findings highlight the urgent necessity of comprehensive health education initiatives focused on enhancing knowledge of disease causation, modes of transmission, signs and symptoms, and potential transmission sites within the community.

Without addressing these fundamental knowledge gaps, efforts to control schistosomiasis face significant obstacles. Thus, investing in educational programs tailored to local contexts is paramount to improving disease understanding, fostering community engagement, and ultimately enhancing the effectiveness of intervention strategies aimed at combating schistosomiasis.

## Data Availability

The data used during analysis are available upon request from the corresponding author

## Abbreviations

MDA: Mass Drug Administration
FGD: Focus Group Discussion
OR: Odd ratio
AOR: Adjusted odd ratio
WHO: World Health Organization
HDSS: Health Demographic Surveillance System
